# Genetic fixity in the human major histocompatibility complex and block size diversity in the class I region including *HLA-E*

**DOI:** 10.1186/1471-2156-8-14

**Published:** 2007-04-12

**Authors:** Viviana Romero, Charles E Larsen, Jonathan S Duke-Cohan, Edward A Fox, Tatiana Romero, Olga P Clavijo, Dolores A Fici, Zaheed Husain, Ingrid Almeciga, Dennis R Alford, Zuheir L Awdeh, Joaquin Zuñiga, Lama El-Dahdah, Chester A Alper, Edmond J Yunis

**Affiliations:** 1 Department of Cancer Immunology and AIDS, Dana-Farber Cancer Institute, Boston, MA, USA; 2 The CBR Institute for Biomedical Research, Boston, MA, USA; 3 Department of Medicine, Harvard Medical School, Boston, MA, USA; 4 Department of Medical Oncology, Dana-Farber Cancer Institute, Boston, MA, USA; 5 Molecular Diagnostics Laboratory, Dana-Farber Cancer Institute, Boston, MA, USA; 6 Department of Pathology, Harvard Medical School, Boston, MA, USA; 7 Instituto Nacional de Enfermedades Respiratorias, Mexico City, Mexico; 8 Department of Medical Genetics, Mayo Clinic, Rochester, MN, USA; 9 Department of Pediatrics, Harvard Medical School, Boston, MA, USA

## Abstract

**Background:**

The definition of human MHC class I haplotypes through association of HLA-A, HLA-Cw and HLA-B has been used to analyze ethnicity, population migrations and disease association.

**Results:**

Here, we present HLA-E allele haplotype association and population linkage disequilibrium (LD) analysis within the ~1.3 Mb bounded by HLA-B/Cw and HLA-A to increase the resolution of identified class I haplotypes. Through local breakdown of LD, we inferred ancestral recombination points both upstream and downstream of HLA-E contributing to alternative block structures within previously identified haplotypes. Through single nucleotide polymorphism (SNP) analysis of the MHC region, we also confirmed the essential genetic fixity, previously inferred by MHC allele analysis, of three conserved extended haplotypes (CEHs), and we demonstrated that commercially-available SNP analysis can be used in the MHC to help define CEHs and CEH fragments.

**Conclusion:**

We conclude that to generate high-resolution maps for relating MHC haplotypes to disease susceptibility, both SNP and MHC allele analysis must be conducted as complementary techniques.

## Background

The human major histocompatibility complex (MHC) is a highly polymorphic genomic region occupying approximately 4 Mb on chromosome 6p21.3. In addition to the major HLA class I and class II gene clusters, there are several other HLA-related and immune response-related genes, some of unknown function, as well as likely pseudogenes. The rich polymorphism in this region is a critical determinant for success in tissue transplantation, and in recent years has found a further use in characterizing both ethnic and geographical population relationships. Haplotype analysis is based on the conservation of short blocks of conserved DNA sequence containing specific allele combinations of two or more adjacent or nearby genetic loci. Within the MHC region, a limited number of specific haplotypes are known to be shared by unrelated individuals of well-defined human populations. These relatively long stretches of conserved DNA sequence in the MHC have been termed conserved extended haplotypes (CEHs) [1] or ancestral haplotypes [2,3]. It is also well recognized that CEHs may be represented as a higher order of association, through successive generations, of four or more defined MHC blocks, showing a stronger linkage disequilibrium (LD) to that expected by random recombination.

Portions of a few CEHs can be detected by maximum likelihood statistics but much more precisely and completely by family studies and direct counting [1-6]. In either instance, LD can be analyzed and a significance assigned to the association [1-6]. MHC haplotype blocks and the larger CEHs are usually inherited intact as a unit, and the allele frequency distribution of particular MHC locus combinations in individuals is non-random [1-7]. Reports describe the existence of blocks of conserved DNA sequence in the range of 5 to 150 kb within the human genome separated by sites of high recombination activity [8-10]. These reports, based on LD analysis applied to single nucleotide polymorphism (SNP) data, suggested the blocks represent relatively uniform lengths of conserved DNA sequence maintained throughout the human population as haplotypes.

Conserved MHC blocks and CEHs have been shown to represent markers of human diversity and/or disease susceptibility [4]. Multi-block conserved haplotypes are not limited to the MHC region since genes encoding drug metabolizing enzymes [11], hormone receptors [12] or microtubule-associated proteins [13] are also associated with extended haplotype blocks. For human MHC studies, past work has focused on haplotypes defined by the relationship of classical HLA class I and class II loci and intermediate MHC genes. The *HLA-E *locus, located approximately halfway between the *HLA-A *and *Cw *class I loci approximately 780 kb telomeric to *HLA-C*, has limited polymorphism and has not generally been incorporated into HLA association studies. Here, we describe newly identified block associations within the MHC, specifically determining the distribution of *HLA-E *alleles in relation to HLA-A, B, Cw, complotype and DRB1 blocks, defining a set of CEHs extending over 2.6 Mb (1.5% of chromosome 6). The inclusion of *HLA-E *in MHC haplotype analysis significantly improves the resolution of class I haplotypic blocks, further refining our ability to analyze associations of the human MHC to disease. Through SNP analysis of the MHC class I/class II region, we confirmed the regional genetic fixity identified by MHC allele analysis and demonstrated that SNPs can be used in the MHC to help define CEHs and CEH fragments.

## Results

To improve human MHC haplotype resolution, we initially set about determining *HLA-E *allele polymorphism in the *HLA-A*/*HLA-Cw *interval. Within our samples, only 4 of the currently-identified *HLA-E *alleles were identified (E*0101, E*010301, E*010302 and E*010303) while HLA-E*0104 was not detected. We did not type for the recently identified allele HLA-E*010304 [14]; our typing method would have designated such an allele, if it existed in our subjects, as HLA-E*010302. HLA-E*010303 was found in only one of 176 individuals screened (representing subjects from all 3 panels studied) and was therefore not tested for in the other subjects, but frequent alleles found were HLA-E*0101, followed by E*010302 and E*010301 in 583 individuals. *HLA-A*, *Cw *and *B *alleles were identified at expected frequencies for Caucasian, African-American and Hispanic populations, respectively [1]. In 216 individuals (Panel 1), we found 9 statistically significant haplotypes between *HLA-A *and HLA-Cw, B, only 5 between *HLA-E *and HLA-Cw/B and 7 between *HLA-A *and *HLA-E *(Table 1). Of the latter, the two most significant were (A*0101, E*0101) and (A*0301, E*010302). Of the 5 identified associations between *HLA-E *and HLA-Cw/B, the most significant were (E*0101, Cw*0701, B*0801) and (E*010302, Cw*0702, B*0702). Analysis of the entire class I region revealed 9 haplotypes, of which the most significant were (A*0101, E*0101, Cw*0701, B*0801); (A*0301, E*010302, Cw*0702, B*0702) and (A*0201, E*0101, Cw*0501, B*4402).

**Table 1:** Statistical analysis of HLA-A, -E, -Cw and -B haplotypes for Panel 1.

**I**				**II**				**III**				
HLA-A*	HLA-E*	**f**^1^	**P**^2^	HLA-E*	HLA-Cw*/B*	**f**^1^	**P**^2^	HLA-A*	HLA-E*	HLA-Cw*/B*	**f**^1^	**P**^2^

***0101***	***0101***	95/432	< 1 × 10^-7^	***0101***	***0701, 0801***	51/432	1.2 × 10^-6^	***0101***	***0101***	***0701, 0801***	42/432	< 1 × 10^-7^
					***0602, 5701***	14/432	ns			***0602, 5701***	10/432	5 × 10^-6^
	***0101***	39/432	ns	***0101***	***0501, 4402***	13/432	ns		***0101***	***0501, 4402***	12/432	< 1 × 10^-7^
***0201***					***07XX, 4402***	16/432	ns	***0201***		***07xx, 4402***	6/432	ns
	***010302***	28/432	0.0138	***010302***	***03xx, 40xx***	10/432	0.006		***010302***	***03xx, 40xx***	7/432	0.0001
***0301***	***010302***	30/432	3 × 10^-7^	***010302***	***0702, 0702***	32/432	< 1 × 10^-7^	***0301***	***010302***	***0702, 0702***	21/432	< 1 × 10^-7^
	***010301***	13/432	0.00213									
***1101***	***0101***	20/432	ns	***0101***	***0401, 3501***	36/432	ns	***0101***	***0101***	***0401, 3501***	6/432	ns
	***010301***	8/432	0.025	***010301***	***0602, 13XX***	6/432	0.0005		***010301***	***0602, 13xx***	3/432	ns
***2301***	***0101***	9/432	ns	***0101***	***08xx, 14xx***	11/432	ns	***2301***	***0101***	***08xx, 14xx***	1/432	ns
					***0401, 4403***	3/432	ns			***0401, 4403***	2/432	0.0154
***2402***	***0101***	31/432	ns	***0101***	***0401, 3501***	36/432	ns	***2402***	***0101***	***0401, 3501***	13/432	0.0008
	***010301***	8/432	ns	***010301***	***12xx, 5201***	2/432	ns		***010301***	***12xx, 5201***	2/432	ns
***24xx***	***010302***	16/432	ns	***010302***	***03xx, 15xx***	9/432	ns	***24xx***	***010302***	***03xx, 15xx***	4/432	ns
***2601***	***010301***	4/432	0.022	***010301***	***1203, 3801***	4/432	0.012	***2601***	***010301***	***1203, 3801***	4/432	2 × 10^-6^
***2902***	***010302***	4/432	ns	***010302***	***1601, 4403***	3/432	ns	***2902***	***010302***	***1601, 4403***	2/432	0.005
***3001***	***010302***	2/432	ns	***010302***	***0501, 1801***	2/432	ns	***3001***	***010302***	***0501, 1801***	1/432	ns
***68xx***	***010302***	14/432	0.0183									

^1^Frequency (f)

^2^Probability (p)

Not significant (ns)

**Figure 1:**
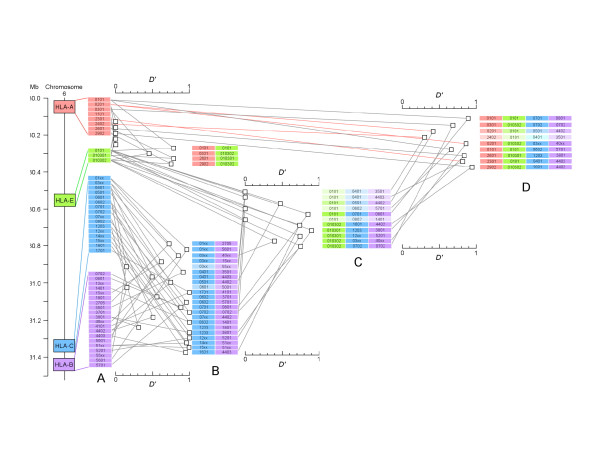
**Linkage disequilibrium (LD) analysis for haplotypes across the human class I region on chromosome 6**. *Column A *depicts individual alleles detected and analyzed within the Panel 2 subjects. The horizontal scale to the right of *column A *presents normalized LD (*D'*) between 2 loci (*HLA-A *and *HLA-E*, and *HLA-C *and *HLA-B*, respectively). To the right of each *D' *value (□), the relevant paired loci with significant association (P < 0.005 in bold color; P < 0.05 in weaker color) are depicted (*column B*). To the right of *column B*, the horizontal *D' *scale depicts the association (□) between *HLA-E *and paired HLA-Cw/HLA-B alleles, with the associated alleles listed in *column C *where strong significant associations (P < 0.005 to < 0.00002) are listed in bold color, weaker associations (P < 0.05) in intermediate color, and non-significant associations indicated by lightest color. To the right of *column C*, the horizontal *D' *scale depicts the association (□) between *HLA-A*, *-E*, *-Cw *and *-B *representing HLA class I haplotypes over 1.4 Mb, where the associated alleles are listed in *column D *(P < 0.0001 in bold, P < 0.002 in intermediate, and P < 0.05 in weak colors). The red lines indicate HLA-A alleles which are not in LD with *HLA-E *upon 2 locus analysis, but become significantly associated in the context of the 4 locus haplotypes including HLA-Cw/HLA-B.

Many significant HLA-Cw/B associations were found within Panel 2, as expected due to the physical proximity of *HLA-Cw *and *-B *(85 kb). Extending the region to 864 kb between *HLA-E *and *HLA-B*, 4 of the same HLA class I haplotypes found in Panel 1 individuals and 4 other statistically significant class I haplotypes were found (Fig. 1, column C). LD analysis of the complete class I region encompassing 1.41 Mb identified the same 9 class I haplotypes found in Panel 1 (Fig. 1, column D). All four HLA-A/E pairs in LD (Fig. 1, column B) were part of at least one of the larger class I haplotypes (gray lines). However, some of the larger haplotypes contained sub-domain regions not strongly linked when analyzed independently. Specifically, 4 HLA-A/E pairs ((A*0201, E*0101); (A*2301, E*0101); (A*2402, E*0101) and (A*0201, E*010302)) found in the larger class I haplotypes (Fig. 1, red lines) did not show significant LD when analyzed alone. Analysis of HLA-E to Cw/B (Fig. 1, column C) revealed that HLA-E*0101 was not in LD with (Cw*0401, B*3501); (Cw*0602, B*5701) nor (Cw*0401, B*4403) despite significant LD when the haplotypes included *HLA-A *(Fig 1, Column D). Conversely, one haplotype with strong D', (E*010301, Cw*12xx, B*5201), was not in LD with *HLA-A*. From these results, we infer ancestral breakpoints both centromeric and telomeric to *HLA-E*.

Within Panel 3, we also studied CEHs, ranging over 2.6 Mb, consisting of their HLA class I loci (Table 2) along with the class II *HLA-DRB1 *locus and the closely-linked complement genes *BF*, *C2*, *C4A *and *C4B *(the complotype; Table 3). *HLA-E *alleles were found to be in significant association with 10 CEHs (Table 3), but excluding *HLA-A *reduced this number. Nevertheless, *HLA-E *association with the Cw/B block and *HLA-A *in Panel 3 (Table 2) showed significance for 7 of the 9 class I haplotypes observed in Panels 1 and 2. Furthermore, 6 other class I haplotypes not found in Panels 1 or 2 had statistical significance in Panel 3.

**Table 2:** Statistical analysis of HLA-A, -E, -Cw and -B haplotypes for Panel 3.

HLA-A*	HLA-E*	*f*	*p*	HLA-E*	HLA-Cw*/B*	*f*	*p*	HLA-A*	HLA-E*	HLA-Cw*/B*	*f*	*p*
***01***	***0101***	61/258	< 1 × 10^-7^	***0101***	***07, 08***	49/258	9 × 10^-6^	***01***	***0101***	***07, 08***	44/258	< 1 × 10^-7^
				***0101***	***06, 57***	12/258	ns	***01***	***0101***	***06, 57***	10/258	5 × 10^-5^
***02***	***0101***	34/258	ns	***0101***	***07, 07***	10/258	ns	***02***	***0101***	***07,07***	7/258	0.007
				***0101***	***05, 44***	9/258	ns	***02***	***0101***	***05, 44***	8/258	0.00013
				***0101***	***06, 50***	6/258	ns	***02***	***0101***	***06, 50***	3/258	ns
***02***	***010302***	15/258	ns	***010302***	***03,15***	2/258	ns	***02***	***010302***	***03,15***	3/258	ns
***03***	***0101***	6/258	ns	***0101***	***06, 47***	2/258	ns	***03***	***0101***	***06, 47***	2/258	ns
***03***	***010302***	16/258	3 × 10^-5^	***010302***	***07, 07***	18/258	0.00046	***03***	***010302***	***07, 07***	14/258	< 1 × 10^-7^
***11***	***0101***	9/258	ns	***0101***	***04, 35***	7/258	ns	***11***	***0101***	***04, 35***	4/258	0.0006
***23***	***0101***	10/258	ns	***0101***	***04, 44***	9/258	ns	***23***	***0101***	***04, 44***	7/258	< 1 × 10^-7^
***24***	***0101***	10/258	ns	***0101***	***08, 14***	7/258	ns	***23***	***0101***	***08, 14***	1/258	ns
***25***	***0101***	5/258	ns	***0101***	***12, 18***	8/258	ns	***25***	***0101***	***12, 18***	5/258	< 1 × 10^-7^
***26***	***010301***	16/258	< 1 × 10^-7^	***010301***	***12, 38***	14/258	< 1 × 10^-7^	***26***	***010301***	***12, 38***	14/258	< 1 × 10^-7^
***29***	***010302***	17/258	9 × 10^-7^	***010302***	***16, 44***	16/258	< 1 × 10^-7^	***29***	***010302***	***16, 44***	16/258	< 1 × 10^-7^
***30***	***0101***	6/258	ns	***0101***	***06, 13***	5/258	ns	***30***	***0101***	***06, 13***	4/258	0.00063
***30***	***010302***	10/258	0.0261	***010302***	***05,18***	12/258	0.0002	***30***	***010302***	***05,18***	10/258	< 1 × 10^-7^
***33***	***0101***	4/258	ns	***0101***	***03, 58***	3/258	ns	***33***	***0101***	***03, 58***	2/258	0.009

^1^Frequency (f)

^2^Probability (p)

Not significant (ns)

**Table 3:** Haplotype statistical analysis for Panel 3, extending haplotypes into the complotype and HLA-DRB1 regions.

HLA-A*	HLA-E*	HLA-Cw*/B*	***Complotype***	HLA-DRB1*	**f**^1^	**P**^2^	HLA-E*	HLA-Cw*/B*	***Complotype***	HLA-DRB1*	**f**^1^	**P**^2^
***01***	***0101***	***07, 08***	***SC01***	***03***	41/256	< 1 × 10^-7^	***0101***	***07, 08***	***SC01***	***03***	48/258	1 × 10^-5^
***01***	***0101***	***06, 57***	***SC61***	***07***	7/256	0.011	***0101***	***06, 57***	***SC61***	***07***	9/258	ns
***02***	***0101***	***07,07***	***SC31***	***15***	7/256	0.003	***0101***	***07,07***	***SC31***	***15***	8/258	ns
***02***	***0101***	***05, 44***	***SC30***	***04***	4/256	ns	***0101***	***05, 44***	***SC30***	***04***	4/258	ns
***02***	***0101***	***06, 50***	***S1C2(1,17)***	***07***	3/256	ns	***0101***	***06, 50***	***S1C2(1,17)***	***07***	4/258	ns
***02***	***010302***	***03,15***	***SC33***	***04***	2/256	ns	***010302***	***03,15***	***SC33***	***04***	2/258	ns
***03***	***0101***	***06,47***	***FC91,0***	***07***	2/256	ns	***0101***	***06,47***	***FC91,0***	***07***	2/258	ns
***03***	***010302***	***07, 07***	***SC31***	***15***	8/256	< 1 × 10^-7^	***010302***	***07, 07***	***SC31***	***15***	9/258	0.022
***11***	***0101***	***04, 35***	***SC30***	***01***	2/256	ns	***0101***	***04, 35***	***SC30***	***01***	4/258	ns
***23***	***0101***	***04, 44***	***FC31***	***07***	5/256	1.1 × 10^-5^	***0101***	***04, 44***	***FC31***	***07***	7/258	ns
***24***	***0101***	***08, 14***	***SC2(1,2)***	***01***	1/256	ns	***0101***	***08, 14***	***SC2(1,2)***	***01***	3/258	ns
***25***	***0101***	***12, 18***	***S042***	***15***	3/256	0.0007	***0101***	***12, 18***	***S042***	***15***	6/258	ns
***26***	***010301***	***12, 38***	***SC21***	***04***	9/256	< 1 × 10^-7^	***010301***	***12, 38***	***SC21***	***04***	9/258	< 1 × 10^-7^
***29***	***010302***	***16, 44***	***FC31***	***07***	11/256	< 1 × 10^-7^	***010302***	***16, 44***	***FC31***	***07***	11/258	1.2 × 10^-5^
***30***	***0101***	***06, 13***	***SC31***	***07***	4/256	0.0007	***0101***	***06, 13***	***SC31***	***07***	5/258	ns
***30***	***010302***	***05,18***	***F1C30***	***03***	10/256	< 1 × 10^-7^	***010302***	***05,18***	***F1C30***	***03***	12/258	0.0002
***33***	***0101***	***03, 58***	***SC30***	***03***	1/256	ns	***0101***	***03, 58***	***SC30***	***03***	2/258	ns

^1^Frequency (f)

^2^Probability (p)

Not significant (ns)

Inclusion of *HLA-E *improved the definition of CEH class I fragments. For example, HLA-E*0101 was a marker for the CEHs [HLA-A*01, Cw*07, B*08, *SC01*, DRB1*07], [HLA-A*30, Cw*06, B*13, *SC31*, DRB1*07], [HLA-A*25, Cw*12, B*18, *S042*, DRB1*15] and [HLA-A*01, Cw*06, B*57, *SC61*, DRB1*07]. Likewise, HLA-E*010301 was a marker for the CEH [HLA-A*26, Cw*12, B*38, *SC21*, DRB1*04] and HLA-E*010302 was a marker for the CEH [HLA-A*30, Cw*05, B*18, *F1C30*, DRB1*03]. Furthermore, *HLA-E *could distinguish class I haplotype variants of at least one CEH: in the two most frequent class I variants of the CEH [HLA-Cw*07, B*07, *SC31*, DRB1*15], HLA-A*02 was associated with HLA-E*0101 while HLA-A*03 was associated with HLA-E*010302. Finally, we found *HLA-E *to be an additional class I locus able to differentiate two HLA-B*4403 CEH variants: [HLA-A*2902, E*010302, Cw*1601, B*4403, *FC31*, DRB1*07] and [HLA-A*2301, E*0101, Cw*04xx, B*4403, *FC31*, DRB1*07].

Panel 3 provided further evidence of ancient recombination within the HLA class I region both centromeric and telomeric to *HLA-E*. The two HLA-A, E variants of the CEH [HLA-Cw*07, B*07, *SC31*, DRB1*1501] strongly suggest a past recombination event between *HLA-E *and *HLA-C*. Conversely, the larger number of (HLA-E*0101, Cw*08, B*14) and HLA-E*0101, Cw*06, B*50) haplotypes found as compared with their most frequent HLA-A variants implies past recombination events between *HLA-E *and *HLA-A*. In summary, we demonstrate, by both χ^2 ^and LD analysis, non-random association of *HLA-E *alleles with alleles at other class I loci and the *HLA-E *allele markers for 10 CEHs. Through breakdown of LD between MHC blocks, we once again infer recombination breakpoints on either side of *HLA-E*. Further, extension of the analysis from *HLA-A *to *HLA-DRB1 *increases the size of allele-defined CEHs up to 2.6 Mb.

**Figure 2:**
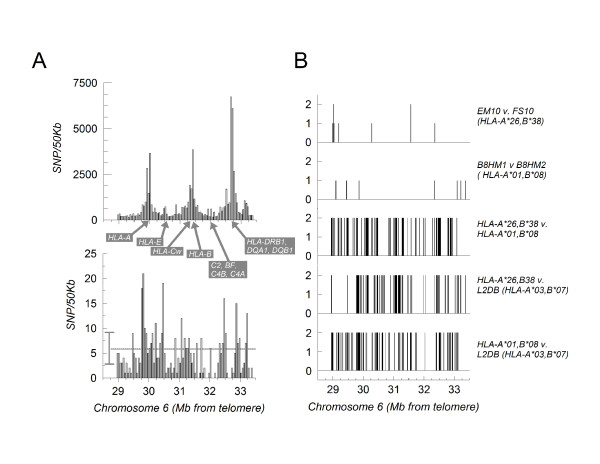
**Comparison of SNPs between CEHs**. A. *Upper panel*: Previously reported SNP distribution over the human MHC (NCBI dbSNP Build 126) indicating the higher density of SNPs in the HLA-A, Cw, B and HLA-DR/DQ regions. Across the indicated region (position 28,944,796 to 33,362,643; from ~1 Mb telomeric to *HLA-A *to ~0.2 Mb centromeric to *HLA-DPB1*), there is a mean frequency of ~790 SNP/50 kb genomic DNA. *Lower panel*: Distribution of SNPs incorporated into the Affymetrix GeneChip Human Mapping 500 K array. The horizontal line represents the mean distribution in the region (~5 SNP/50 kb) and the vertical bar to the left of the line indicates one standard deviation. Thus, the chip uses only 0.6% (428 SNPs) of the SNPs reported in the region to date. Note that gene chip SNP representation in the regions harboring defined HLA polymorphic alleles is even lower. B. *Top panel*: SNP variations were determined between two cell lines (EM10 and FS10) homozygous for the CEH [HLA-A*2601, E*010301, Cw*1203, B*3801, *SC21*, DRB1*0402, DQA1*0301, DQB1*0302] except for EM10, which is heterozygous for HLA-E*0101 and HLA-E*010301. For all panels in this figure, a value of 0 indicates homozygous identity between the two cell lines, a value of 1 indicates heterozygosity for the SNP in at least one of the cell lines, and a value of 2 indicates complete discordance between the two cell lines. *Second panel*: SNP variations between the B8HM1 and B8HM2 cell lines each homozygous for the CEH [HLA-A*0101, E*0101, Cw*0701, B*0801, *SC01*, DRB1*0301, DQB1*0201]. *Third panel*: A comparison of the homozygous SNP identities shared between EM10 and FS10 (the HLA-A*26, B*38 CEH) cell lines with the homozygous SNP identities shared between B8HM1 and B8HM2 (the HLA-A*01, B*08 CEH) cell lines. Only those SNPs for which there was an unequivocal call for all four cell lines were included. *Fourth panel*: SNP variations between the HLA-A*26, B*38 CEH and the L2DB cell line homozygous for the independent HLA-A*03, B*07 CEH ([HLA-A*0301, E*010302, Cw*0702, B*0702, *SC31*, DRB1*1501, DQA1*0102, DQB1*0602]. *Bottom panel*: SNP variations between the HLA-A*01, B*08 CEH and the L2DB cell line homozygous for the HLA-A*03, B*07 CEH.

LD analysis of SNP databases derived from the general population has gained widespread credibility as an alternative means for tracing evolutionary human genetic networks [8]. Within the human MHC spanning ~4.5 Mb, the frequency distribution of identified SNPs (71,136 deposited in NCBI dbSNP Build 126) is highly non-uniform where regions with peaks represent regions of highly polymorphic HLA-associated genes (Fig. 2A, upper panel). The currently available gene mapping chips sample 500,000 SNPs for whole genome mapping with only 428 SNPs within the MHC region (Fig. 2A, lower panel). This limited sampling (0.6%) is further affected by the non-uniform representation of SNPs on screening arrays where the defined HLA genes are under-represented (because sampling has no significance unless the selected SNPs are to be screened within a preselected limited set of haplotypes). Accordingly, gene array SNPs tend to exclude the highly polymorphic HLA class I and class II regions. CEHs and genetic fixity, however, have been defined by alleles of the coding genes. We therefore used SNP analysis to study the co-segregation implied by locus allele analysis.

A comparison of SNPs in cell lines homozygous for major HLA loci was performed. Except for *HLA-E*, where the EM10 cell line is heterozygous for HLA-E*0101 and HLA-E*010301, EM10 and FS10 are homozygous for the CEH [HLA-A*2601, E*010301, Cw*1203, B*3801, *SC21*, DRB1*0402, DQA1*0301, DQB1*0302] found at high frequency in Ashkenazi Jews [4]. Over the entire MHC region representing 369 individual SNPs which could be reliably identified in both cell lines, only 5 instances of heterozygosity in either cell line and only 2 instances of complete discordance between the two cell lines were detected (1.9%; Fig. 2B), thus supporting the block linkage implied by our other gene analyses (Tables 1, 2, 3 and Fig. 1). Further confirming our supposition concerning the relationship between actual SNPs to those selected on the chip array, the heterozygosity at the HLA-E locus of the EM10 cell line was not detected by SNP analysis.

Similar SNP analysis of 2 cell lines (B8HM1 and B8HM2) homozygous for the most frequent CEH in American and British Caucasians [4] ([HLAA*0101, E*0101, Cw*0701, B*0801, *SC01*, DRB1*0301, DQA1*0501, DQB1*0201]) revealed only 7 instances of heterozygosity and no instances of complete discordance between the two cell lines of 370 unambiguous SNPs analyzed (Fig. 2B). Selecting only those SNPs identical in EM10 and FS10 and designated "HLA-A*26, B*38", and comparing them with those SNPs identical in B8HM1 and B8HM2 designated "HLA-A*01, B*08" (Fig. 2B), we observed 113/271 (41.7%) complete discordance between the two sets (p: < 1 × 10^-7^). To demonstrate that the striking similarity of the SNPs in EM10 as compared with FS10 and in B8HM1 as compared with B8HM2 and that the striking difference between the HLA-A*26, B*38 and HLA-A*01, B*08 SNPs were not anomalies of the cell lines chosen, both sets of SNPs were independently compared to those of another cell line (L2DB), which is homozygous for a different CEH ([HLA-A*0301, E*010302, Cw*0702, B*0702, *SC31*, DRB1*1501, DQA1*0102, DQB1*0602]; designated "HLA-A*03, B*07"). As shown in Fig. 2B, the HLA-A*03, B*07 CEH SNPs differ significantly from those of either the HLA-A*26, B*38 (31.25% complete discordance; p: < 1 × 10^-7^) or the HLA-A*01, B*08 (35.1% complete discordance; p: < 1 × 10^-7^) CEHs.

## Discussion

Human MHC polymorphisms likely represent the geographic dispersal of early man and expansion of limited haplotypes in concert with selection driven by local microbial organisms. This has led to association of haplotypes with both ethnicity and various immunopathologies. It has been postulated that the basis for some of the disease-associations may be a cross-reactivity between a microbe-specific peptide sequence and a closely-related host sequence leading to anti-host reactivity (e.g., HLA-B27 and ankylosing spondylitis [15]). To accurately identify the relationship of a genetic locus to disease, it is critical to determine whether an allele is associated with such pathology or whether the locus is co-segregating due to proximity with the responsible gene. Consideration of co-segregation is particularly critical given that direct determination of MHC haplotypes from family studies shows frequently occurring small block variants and given that a third to a half of Caucasian haplotypes are fixed from *HLA-B *to HLA-DRB1/DQB1 (at least 1 Mb) as CEHs [1-6].

To increase the resolution of haplotypes within the human MHC region defined by population LD analysis, this study was initially conceived as a means of incorporating *HLA-E *into the other class I, class II and complotype regions. HLA-E is an HLA-1b-type molecule of limited polymorphism interacting with natural killer receptors, functioning as an important mediator of cytotoxicity [16-18]. Initial LD analysis suggested that *HLA-E *polymorphism occurred early in hominid development and stabilized in *Homo sapiens *before the major geographic dispersals [19]. Consequently, it seems likely that the distribution of *HLA-E *alleles represents population migration with inbred expansion. In support of this notion, our analysis of *HLA-E *alleles identified 3 alleles, (HLA-E*0101, HLA-E*010301 and HLA-E*010302), non-randomly associated with particular CEHs. Our typing method was not designed to detect the recently identified allele HLA-E*010304 [14], and, if it had been present in any of the haplotypes, it would be reported here as HLA-E*010302. We are unaware of any report describing the population frequency of that allele; we shall clarify its presence or absence in particular CEHs in future studies. We identify apparent ancestral breakpoints upstream and downstream of *HLA-E*, and in the context of the limited number of *HLA-E *alleles identified, this would seem to reinforce the notion of *HLA-*E polymorphism occurring early in hominid development and stabilizing, and thus not in conflict in any way with the more recent stabilization of extended haplotypes confirmed here both by population LD analysis and SNP analysis. There is the further implication that recombination breakpoints in the HLA region are relatively infrequent.

Haplotype blocks and breakpoints revealed by population analysis do not always correlate with those identified by direct haplotype sequencing of sperm [20-22]. Sperm crossover points may indicate the potential for recombination while family studies represent the practical end result reflecting fertilization potential and environmental selective pressures. Accordingly, recombination frequencies from a single individual or limited pool should be used cautiously to describe the effect of recombination on haplotype frequencies in the population [6]. Other suggested mechanisms to explain discrepancies between sperm crossover points and family-inferred breakpoints include higher crossover rates in female gametes not observed in sperm [21], as well as the possibility that some breakpoints recognized by segregation analysis represent inactive ancestral recombination "hot spots" which have become fixed in populations [20].

Since selection in its most accepted formulation operates mostly upon protein products, the power of allele variant haplotype analysis is undeniable. In recent reports, extensive analysis of single nucleotide polymorphisms (SNP) has been used to produce high-resolution maps of breakpoints at greater frequency identified by allele variant population haplotype analysis. Some have argued that allele variant segregation and population haplotype analysis is erratic, influenced by gene frequency and population dynamics [23]. On the contrary, it is exactly these properties that have allowed allele variant population haplotype analysis to identify ethnic descent and migration of *Homo sapiens *so precisely.

LD analysis of SNP distribution in haplotypes defined by maximum likelihood methods has revealed genomic structures similar to and yet far less complex than those identified by allele variants haplotyped by segregation analysis [1-6,24]. The former method may be responsible for some oversimplification of recent haplotype analyses [1,4], but using SNP markers alone may also pose inherent problems. High-throughput localization of SNP distribution is inarguably efficient, but the vast majority of SNPs reside outside coding regions. Although there is potential for polymorphisms in non-coding promoter and intron DNA to influence subsequent transcription and splicing of a gene [25,26], selection pressure is more likely to operate at the protein level. Particular haplotype block combinations of relatively long genomic distance are likely to have been initially fixed in response to geographical or environmental influences. The passage of time, migration and alterations in climate and local flora prevent analysis, but identification of other non-immune-related haplotype blocks offers support for selection influence on haplotype structure [11]. However, a recent report "mapping" the MHC using both HLA alleles and SNPs by LD analysis of haplotypes defined by maximum likelihood methods [24], suggests that the primary reason such maps fail to detect the details of human population haplotype structure [1-6] is their use of probabilistic (as opposed to segregation) analysis.

## Conclusion

The identified associations of *HLA-E *alleles and SNPs within established CEHs, increase the extent of their recognized fixity. For example, HLA-B*4403 distributes with two CEH class I variants, (HLA-A*2301, Cw*04xx, B*4403) (with two HLA-Cw*04xx variants of its own) and (HLA-A*2902, Cw*1601, B*4403) [27]. *HLA-E *allele identification improves the class I differentiation of these CEHs to (HLA-A*2301, E*0101, Cw*04xx, B*4403) and (HLA-A*2902, E*010302, Cw*1601, B*4403), respectively. Results of several recent studies on two specific CEHs support our general conclusion of the fixity of CEHs in the class I region. Both high density SNP [28] and resequencing [29] analysis of the A1-B8-DR3 CEH and high density SNP analysis of the A30-B18-DR3 CEH [30] showed the essential sequence fixity of each of those haplotypes in unrelated individuals. Here, in a more limited set of samples, our high density SNP analysis confirms the essential fixity of the CEH [HLA-A*26, Cw*12, B*38, *SC21*, DRB1*04].

**Figure 3:**
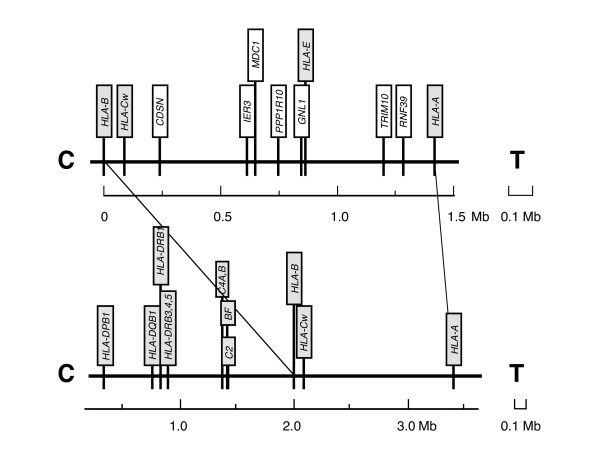
**Human MHC class I map showing known polymorphic genes**. Distances are drawn to scale (see legend), but these may vary at many locations in different haplotypes as a result of limited polymorphic DNA insertions, deletions or gene duplications. Two non-classical polymorphic candidate genes (white text boxes), *RNF39 *(ring finger protein 39) and *TRIM10 *(tripartite motif-containing 10), are located between the HLA-type genes (gray text boxes) *HLA-A *and *HLA-E*, while other polymorphic genes are located between *HLA-E *and *HLA-C*: *CDSN *(corneodesmosin), *IER3 *(immediate early response 3), *MDC1 *(mediator of DNA damage checkpoint 1), *PPP1R10 *(protein phosphatase 1, regulatory inhibitor subunit 10) and *GNL1 *(guanine nucleotide binding protein-like 1). Gene locations are drawn to scale and were taken from the Sanger Institute MHC list for the COX cell line [41] and the distance (in megabases (Mb)) from HLA*B at the centromeric (C) end to an arbitrary point telomeric (T) to *HLA-A *are shown.

Since the SNP data so strongly support the genetic fixity of CEHs first observed by direct allele analysis, several approaches may be taken to improve haplotype definition. First, to define the SNP variants of particular CEHs, the density of SNP analysis can be raised to almost complete levels by choosing the limited subset expressed within a predefined CEH. An alternate approach based on the strong SNP support for CEHs, is to identify other polymorphic MHC genes, particularly in the HLA-A to HLA-C region, for consideration in LD analysis. Therefore, we identified several polymorphic markers within the 1.3 Mb of genomic DNA between HLA-A and HLA-C (Fig. 3). Analysis of these markers permits determination of hierarchical haplotype block associations where block variation within the CEH may provide further insights into human diversity and disease susceptibility. Determining the frequency of sizes of DNA blocks in different populations will add a new dimension in the studies of human diversity and gene localization in diseases associated with the MHC class I region [1]. In this latter instance, the high resolution allele analysis will lead to better definition of the associative levels of MHC DNA blocks, CEHs and their fragments influenced by genetic admixture allowing more precise elucidation of disease-associated HLA alleles when comparing different ethnic groups and nationalities.

## Methods

### Population

All participants either provided clinical samples prior to hematopoietic cell transplantation or gave informed consent for research purposes in accordance with the CBR Institute for Biomedical Research (CBRI) or Dana-Farber Cancer Institute (DFCI) Institutional Review Board-approved protocols. The initial panel (Panel 1) was composed of 216 healthy unrelated North American residents typed for *HLA-A*, *HLA-B *and *HLA-C*, of which 56 were homozygous for both *HLA-A *and *HLA-B*, 58 were homozygous for *HLA-B *and heterozygous for *HLA-A*, and 102 were homozygous for *HLA-A *and heterozygous for *HLA-B*. Panel 2 was composed of 176 unrelated parents of 88 Caucasian families. We assigned haplotypes by inheritance.

Panel 3 consisted of three groups of individuals enriched in previously defined MHC CEHs or their markers. The first group were unrelated subjects who provided samples used to generate 25 International Histocompatibility Workshop (IHW) and 5 locally produced cell lines. The second subject group for this panel consisted of 130 subjects in 49 unrelated families whose MHC haplotypes were defined by segregation analysis. The third group consisted of 31 unrelated subjects.

### Cell lines

The EM10, FS10, B8HM1, B8HM2 and L2DB cell lines were used to represent homozygous haplotypes in Figure 2, as described previously [31].

### MHC typing

Genomic DNA was obtained from peripheral blood mononuclear cells (PBMC), EDTA-treated plasma or lymphoblastoid cell lines and was isolated using the QIAamp DNA mini kit (Qiagen, Valencia, CA). Molecular typing of IHW cell lines was previously known [32] and/or was conducted as described below. Molecular typing of samples from Panels 1 and 2 was performed by PCR and sequence-specific oligonucleotide probes (PCR-SSOP) at intermediate to high resolution [33]. SSP molecular typing of non-IHW cell line samples from Panel 3 was performed either using an SSP UniTray kit (Invitrogen/Dynal/Pel-Freez, Brown Deer, WI) or by PCR-SSOP (HLA Quick-Type kits, Lifecodes, Stamford, CT), according to previously described amplification conditions [33]. Some samples from the CBRI had several HLA types identified serologically [34]. Typing of *BF*, *C4A *and *C4B *alleles was done by agarose gel electrophoresis and immunofixation of their protein products with specific antisera, and *C2 *alleles were determined by isoelectric focusing of serum samples in polyacrylamide gels followed by a C2-sensitive hemolytic overlay [35]. MHC complement gene haplotypes or complotypes are designated by their *BF*, *C2*, *C4A*, and *C4B *alleles, in that arbitrary order [7]. Null or *Q0 *alleles are simply designated 0. Thus, FC31 indicates the complotype BF*F, C2*C, C4A*3, C4B*1. Some of the non-*HLA-E *typings have been published previously [4,5,27].

### HLA-E typing

***Amplification ***– After extraction of genomic DNA, published primers were used for the amplification of exons 2 and 3 of the *HLA-E *gene [36]. Amplification reactions were carried out in 50 μl final volume containing 100 ng of genomic DNA, 0.3 mM of each dNTP (Amersham Pharmacia Biotech Inc.), 1× Buffer (Roche Molecular Biochemicals), 1.5 mM MgCl_2_, 1.25 units of *Taq polymerase *(Roche) and 15 pmol of primers. PCR conditions were: 94°C denaturation for 5 min, followed by 35 cycles of 94°C for 1 min, 58°C (exon 2) or 60°C (exon 3) for 1 min, 72°C for 2 min, followed by a final extension step at 72°C for 10 min. Products were visualized by staining with ethidium bromide on 1.8% agarose gels. We did not amplify the region of exon 4 that would have distinguished HLA-E*010304 [14] from the other alleles. ***PCR-SSOP ***– The alleles HLA-E*0101, E*010301, E*010302, and E*0104 were assigned using a PCR-SSOP method as described previously [37]. All the typings included those 4 known internal controls. Briefly, 3 μl of PCR products were blotted onto nylon membranes and dried at room temperature. Denaturation of the DNA on the membranes was performed in constant gentle agitation with 0.4 M NaOH for 10 min and equilibrated in SSC for 5 min. Membranes were dried at room temperature and then illuminated with a 254 nm ultraviolet lamp for 5 min to fix the nucleic acid. Pre-hybridization consisted of incubation with 0.2 ml/cm^2 ^of hybridization buffer (SSC, 1% Blocking Reagent (Roche), 1% N-lauryl sarcosine, 0.02% SDS) and left for 30 min at 42°C. Hybridization was performed at 42°C for 3 hr using new hybridization buffer (0.2 ml/cm^2^) containing oligonucleotides specific for *HLA-E *previously labelled with dig-ddUTP (Roche) [36] followed by two washes in SSPE, 0.1% SDS at room temperature for 5 min each time, washing in 50 ml preheated tetramethylammonium chloride/0.1% SDS solution (Lifecodes Corporation) at 59°C for 20 min and two final washes 50 ml of 2 × SSPE at room temperature for 10 min each time. Membranes were equilibrated in buffer 1 (100 mM Tris-HCl, pH 7.2, 150 mM NaCl) for 5 min followed by blocking in buffer 1 containing 2% blocking reagent (Roche) for 1 hr followed by the detection agent, anti-digoxigenin-AP antibody (75 mU/ml in buffer 1 (Roche) for 30 min. After washing two times in buffer 1 for 15 min each followed by buffer 2 (100 mM Tris-HCl pH 9.5, 100 mM NaCl, 50 mM MgCl_2_) for 5 min, signal was developed by placing wet membranes in pre-warmed Lumiphos (Lifecodes) on acetate sheets, excess solution was removed, and after incubation at 37°C for one hour, the chemiluminescent signal was detected by film exposure. ***PCR-RFLP ***– In a limited number (176) of subjects representing all 3 subject panels, the PCR-RFLP method was used to detect HLA-E*010303. Following amplification of exon 3 as described above, the product was digested with *Bgl1 *(New England Biolabs), separated on a 2.5% agarose gel and stained with ethidium bromide. The presence of HLA-E*010303 was detected by the presence of a specific 247 bp band and a separate 73 bp band, while other alleles yielded bands at 135 bp and 112 bp and the 73 bp band.

### HLA-E haplotype assignment

Panel 1 haplotypes were unambiguously assigned from individuals homozygous for at least *HLA-A *and *HLA-E *(in whom HLA-Cw, B blocks were assigned based on known associations [1]) or homozygous for at least *HLA-E *and HLA-Cw, B. Panel 2 haplotypes were assigned by family study using segregation analysis [5]. For the third panel, we assigned *HLA-E *alleles to 258 MHC haplotypes. Of these, 167 haplotypes (65%) were unambiguously assigned by one of four methods: a) in IHW or locally-produced MHC homozygous cell lines; b) by segregation analysis in pedigrees [5]; c) to previously defined (by segregation analysis) haplotypes in subjects homozygous for *HLA-E*; or d) to deduced haplotypes in subjects homozygous for at least *HLA-E *and their HLA-Cw, B blocks. The cell lines (a, above) were assumed to be consanguineous (and received only one haplotype assignment) unless known not to be consanguineous. At the end of this first analysis, we assigned *HLA-E *alleles to the six most frequent CEHs (Table 3). The remaining haplotypes (n = 91) were assigned *HLA-E *alleles with two assumptions. First, individuals who had all of the class I to complotype markers of at least one CEH were included in the analysis, and all of the markers of a given CEH were assigned to one of the haplotypes. Second, for individuals without clear *HLA-E *assignment (e.g., a family in which all subjects were *HLA-E *heterozygous and identical or an *HLA-E *heterozygous individual without relatives in the study), but who had at least one haplotype with the class I markers of one of the six CEHs defined above, the defined *HLA-E *assignment was given to that CEH.

### SNP analysis

Genomic DNA was digested with *Nsp1 *or *Sty1 *prior to adapter ligation, amplification, end-labeling and hybridization to a GeneChip (GeneChip Human Mapping 500 K Array Set; Affymetrix, Santa Clara, CA). Arrays were analyzed on a GeneChip Scanner 7000 RG and data analyzed using the GTYPE software all according to the manufacturer's directions. 428 SNPs from the region from position 28,944,796 (near the gene *TRIM27*, approximately 1.0 Mb telomeric to *HLA-A*) to 33,362,643 (near the gene *B3GALT4*, approximately 0.2 Mb centromeric to *HLA-DPB1*) were analyzed (Genbank dbSNP build 126 rs209163 to rs466384). In several instances, a clear call on the polymorphism could not be made in which case the SNP was not used. Consequently, depending on the calls for each cell line, approximately 370 SNP with high confidence calls for each cell line were compared (Fig. 2B).

### Statistical analysis

Allele frequencies of HLA generic and allele types were calculated for each of the three panels separately by direct counting [1-6]. LD for alleles at loci between *HLA-E *and *HLA-A *or between *HLA-C *and *HLA-B *was analyzed in Panel 2 using delta (Δ) and normalized delta (D'). Other two-point LD calculations were made between *HLA-E *and the HLA-Cw/B block, with the latter analyzed as a single entity, and between *HLA-A *and HLA-E/Cw/B, with the latter analyzed as a single entity. Although D' normalizes for allele frequency, it does not compensate for sample size. Accordingly, we used Fisher's exact test to provide an additional measure of significance of association of the loci. We defined significant LD as positive normalized delta (D') in the context of p < 0.05. LD is defined as a frequency of possible association for specific alleles at two or more loci (i.e., a putative haplotype) that departs from expectation based on the known frequencies of the individual alleles comprising that haplotype (determined in this report by pedigree (i.e., genotypic data) analysis). In a homogenous population at genetic equilibrium, if the alleles A and B at two loci with frequencies f(A) and f(B), respectively, are completely randomly associated with one another, they form an AB haplotype with a frequency of f(AB) = f(A) · f(B). If these conditions are not met, the alleles are said to be "in LD." The extent of LD is given by Δ = f(AB) - [f(A) · f(B)], in which larger delta (Δ) values indicate greater LD. The LD of a two-locus haplotype, A_i_B_j _will be:

LD (A_i_B_j_) = HF (A_i_B_j_) - a_i_b_j_

where HF is the haplotype frequency and a_i _and b_j_, the frequencies of A_i _and B_j _alleles [1]. The Δ value is converted to a normalized LD value (D') to determine the relative LD irrespective of individual allele frequencies. This normalized value is calculated as:

D' = Δ/Δ_max_

where Δ_max _is the maximum LD value possible [38]. The significance of all the results (Tables 1, 2, 3 and Figure 1) was assessed with Fisher's exact test with Bonferroni correction [39]. Odds ratios (ORs) were calculated with a 95% CI [36].

### MHC gene location and distances

Physical distances between MHC genes were found at the Wellcome Trust Sanger Institute Human Chromosome 6 website [40].

## Abbreviations

CEH: conserved extended haplotype

SNP: single nucleotide polymorphism

LD: linkage disequilibrium

SSOP: sequence-specific oligonucleotide probes

OR: odds ratio

SSP-PCR: sequence-specific primer-PCR

## Authors' contributions

VR performed research, analyzed data and wrote the manuscript. CEL participated in study design and coordination, contributed and analyzed data and wrote the manuscript. JSD-C analyzed data and wrote the paper. EAF, TR, OPC, DAF, IA, DRA and LE-D performed research. ZH analyzed data and cell line CEHs. ZLA and CAA contributed data and cell lines. JZ analyzed data. EJY conceived of the study, participated in its design and coordination and wrote the manuscript. All authors read and approved the final manuscript.
